# The feasibility and safety of one-shot dilatation compared to conventional sequential dilatation in tubeless percutaneous nephrolithotomy: a prospective randomized controlled study

**DOI:** 10.1007/s00240-022-01383-6

**Published:** 2022-12-01

**Authors:** Waleed Ghoneima, Mohamed Makki, Mohamed Amr Lotfi, Amr Mostafa, Amr Elkady, Ahmed M. Rammah

**Affiliations:** Department of Urology, Cairo University Hospitals, Cairo, Egypt

**Keywords:** Tubeless percutaneous nephrolithotomy, One-shot dilation, Sequential dilation

## Abstract

To study the feasibility and safety of One-Shot Dilatation (OSD)**,** versus serial sequential dilatation in tubeless Percutaneous Nephrolithotomy (PCNL). One Hundred and Fifty patients were randomised into two groups; Group A (One-Shot Dilatation), Group B (Serial Dilatation). Twenty-one patients were excluded from the study. Detailed history was taken and full physical examination was performed. Pre-operative routine laboratory investigations were done. Also, non-contrast Computed Tomography of the Urinary Tract (CTUT) and plain urinary tract x-ray were done. Intra-operative assessments of dilatation, total operative, total fluoroscopy and fluoroscopy during dilatation durations were recorded, as well as estimated blood loss. Post-operatively haemoglobin, creatinine levels and CTUT were performed for all patients. Complications, as urinary leakage time, analgesic requirements and hospitalization time were measured. There were statistically significant differences in the intraoperative durations, where Group A had shorter dilatation time, fluoroscopy time during dilatation and total operative time. Group B had a higher complications rate than Group A; 37.9%, 11.3%, respectively. Also, Group B showed haemoglobin drop by 0.44 mg/dl higher than Group A. More doses of analgesia were required for Group B. Hospitalization time and rate of urinary leakage were both in favour of Group A. For patients undergoing Tubeless PCNL, we have concluded that one-shot dilatation seems to be a safer and more feasible technique than Serial dilatation.

## Introduction

Nephrolithiasis is one of the most common diseases of the urinary tract, causing patients to have wide range of clinical presentations, ranging from bothersome symptoms to significant complications and co-morbidities. Management of nephrolithiasis includes variety of options, from conservative measures such as urinary alkalinisation, to open nephrolithotomy. However Percutaneous Nephrolithotomy (PCNL) became the most favourable procedure for large stones, as it has higher stone-free rate, compared to Extracorporeal Shock-Wave Lithotripsy (ESWL) with less complications compared to open approach [1]. In the past few years there have been many modifications in PCNL, besides minimizing the percutaneous access calibre to reduce morbidity regarding parenchymal damage and risk of haemorrhage. Nowadays, many surgeons resort for Tubeless PCNL, where nephrostomy tube is not placed at the end of the procedure, especially when the operation is uneventful [2]. Another modification has been recently preferred by urologists, is the One-Shot Dilatation (OSD) in renal access tract dilatation, where after the insertion of two 0.038″ guidewires (safety and working guidewires), a central Alken with a 30fr Amplatz dilator and the plastic sheath are inserted on the working guidewire. This technique has more advantages, as less operative time, less x-ray exposure time, minimal post-operative haemorrhage, compared to conventional renal access dilatation using sequential metal dilators [3].

## Aim of study

The aim of this work is to study the efficacy and safety of one-shot dilatation in tubeless PCNL (Percutaneous Nephrolithotomy), compared to the conventional sequential dilatation in a prospective randomised controlled study.

## Patients and methods

This study is a prospective randomised controlled study that was conducted in Cairo University Hospitals in the period from March 2020 till February 2021.

### Sample size

Based on the previous work by Frattini et al. 2001 [4] where both types of dilatations were compared in a prospective randomised controlled study, the difference in post-operative decrease in haemoglobin level between the two groups was 0.36 ± 0.67 mg/dl. Using a power of 80 and 5% significance level we were needed to study at least 55 in each group. Sample size calculation was achieved using PS: Power and Sample Size Calculation Software Version 3.1.2 (Vanderbilt University, Nashville, Tennessee, USA).

### Patient confidentiality

A written and verbal consent was obtained from all patients included in this study, explaining the indication for the procedure, its possible risks and complications, and the potential benefits from the study.

### Patient selection

This study was randomised by simple randomization using closed 150 envelopes, divided to two equal groups. Patients were divided into two groups; A & B, where group A included patients for One-Shot dilatation, while, group B, involved the patients for conventional serial Alken dilatation. A random envelope was picked before each case by the senior registrar and the operating surgeon was informed of the result. All adult patients scheduled for tubeless PCNL were admitted in Cairo University Hospitals, in the period from March 2020 till February 2021.

### Inclusion criteria

Patients between 18 and 60-year-old of both genders with clinical & radiological diagnosis of renal stone burden of more than 1.5 cm, or lower calyceal stones more than 1 cm. elected for Tubeless PCNL, were included in the study.

### Exclusion criteria

Patients who had nephrostomy placed at the end of the operation, renal impairment, multiple punctures done in the same operation, bleeding disorders, pregnancy, ectopic or malrotated kidney, skeletal deformities or ipsilateral renal tumour were excluded.

### Data collection

A detailed urological history taking and examination were done for all patients. Preoperative investigations including; complete blood count, coagulation profile, liver and kidney function tests, blood grouping, and urine culture and sensitivity were asked. Urinary tract infections were treated with antibiotics according to the urine culture. Pelvi-abdominal ultrasound, plain UT x-ray and non-enhanced CTUT were done to all patients. BMI (Body Mass Index) was calculated. Data regarding the stone site, size and density (in Hounsfield units) were noted as seen in the CT scan. Also, parenchymal thickness (between renal capsule and the targeted calyx) and skin to stone distance were measured in the CT scan by the radiologist, measuring it from the point of the largest stone diameter at a 45° angle from the horizontal line in the axial view using the Marosis view. Thirty minutes prior to anaesthetic induction, patient was given a single shot of antibiotic according to the urine culture and sensitivity test. Patients with asymptomatic bacteriuria or patients with negative urine culture were given intravenous cephalosporins.

After induction of anaesthesia, ureteric catheter was inserted using cystoscopy. The patient was then placed usually in prone position (supine position in some rare cases such as very obese and asthmatic cases whose prone position would compromise the ventilation parameters). A retrograde pyelogram was done to decide which calyx to puncture. The kidney was then punctured under fluoroscopic guidance carefully using an 18G puncture needle, then a J-tip guidewire was inserted through the needle. The puncture needle is removed and replaced with a reversed central Alken dilator with the smallest Alken dilator was placed over it, then the central Alken was removed to place a second ‘safety’ guidewire. The central Alken was placed over the ‘working’ guidewire then according to the dilatation method allocated, if serial dilatation was chosen, then conventional dilatation using telescopic metal Alken dilators (Karl Storz Telescopic bougie set 27090 A 9-24Fr Germany) was done. If One-Shot dilatation was chosen then a 30 Fr Amplatz dilator (30 Fr single use Amplatz dilator Boston Scientific with renal sheath M0062601120 USA) was placed over the central Alken and dilated carefully into the desired calyx with fluoroscopic guidance. Amplatz sheath was placed over the dilator. The nephroscope (Karl Storz nephroscope 27092 AMA, 19 cm with sheath 27093 CD 24Fr Germany) was then introduced to confirm successful intra-renal access and to view the stone(s), which was then dealt with accordingly. At the end of the procedure, all guide wires and access sheath were removed and the wound was sutured. Ureteric stent was fixed in patients with either residual stones, renal pelvic perforation or post-operative persistent pain, fever or leakage. Intraoperatively, the total operative time was measured from the time of anaesthetic induction to the end of the procedure, after removing the Amplatz sheath. Also, total fluoroscopy time during the whole procedure was recorded from the fluoroscopy unit. More specifically two more timings were recorded to avoid bias; dilatation time or renal access time measured starting from after placing the central Alken rod over the guide wire, till after dilatation when the Amplatz sheath is placed inside the calyx. Also, fluoroscopy time during dilatation was recorded from the fluoroscopy unit during that same duration. Post-operatively, periodic vital signs and temperature were measured and abdominal examination was done to exclude signs of rigidity. Urine output and colour were monitored throughout the hospital stay. Wound dressing was periodically inspected for signs of leakage and once it became dry, the ureteric catheter was removed. Usually, the ureteric catheter was removed after 24 h unless there was no urine leakage. Time taken for the wound to be dry was noted. Documentation of the post-operative pain was done using a pain scale from 0 to 10, where 0 means no or negligible pain and 10 correlates to maximum pain perceived by the patient, also the frequency of analgesia (NSAIDs) given to the patient was noted. Complications were documented and classified according to the Clavien-Dindo classification. Post-operative haemoglobin concentration and serum creatinine level were measured. Blood transfusion was administered when the haemoglobin drop was less than 9 g/dl or hypotension despite fluid resuscitation. A non-contrast CTUT was done on post-operative day 1 to assess the possibility of any residual stones present and noted if they were larger than 5 mm. Residual stone burden was measured as total of their maximum diameter using post-operative CTUT. Hospitalization time, since the operation was documented in hours. The patient was eligible for hospital discharge when he was vitally stable, no active haematuria, regained normal bowel habits and no significant pain. Patients received the follow-up timing schedule to do abdominopelvic ultrasound and urine analysis.

## Results

In the course of this study, 150 patients were elected for Tubeless PCNL who were matched with the inclusion criteria and shared none of the exclusion criteria. Patients were randomly divided into two equal groups. Group A (OSD PCNL) and group B (Serial Dilatation PCNL). During the study 21 patients were excluded from the study, 4 patients were from Group A, 17 patients were excluded from Group B, either due to multiple punctures for large stones or missed follow up. A total of 129 patients were included in this study, 71 from Group A and 58 patients from Group B.

### Patient demography

The mean age for both groups was is 43.8 ± 12.1 years, where it was 43.2 ± 13.0 years for Group A, and 44.6 ± 11.1 years for Group B. Regarding the gender demography of both groups; most of the patients included in the study were males. No significant difference between both groups regarding patients who underwent previous ipsilateral renal surgery for past stones (Open or endoscopic) or ESWL. There was no statistical difference regarding mean BMI for both groups.

### Stone parameters

**Fig. 1:**
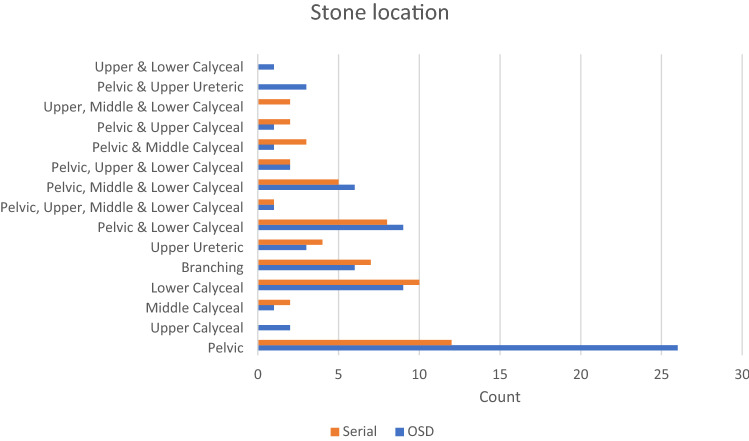
Illustrative clustered bar chart demonstrating different sites for stones for both groups

The mean number of stones for all patients was 1.81 ± 1.08 stones; 1.63 ± 0.989 stones for Group A and 2.03 ± 1.15 stones for Group B (p = 0.03). There was no significant difference between both groups regarding the stone burden for both groups which was 2.7 ± 1.1 cm (2.64 ± 1.15 for Group A and 2.78 ± 1.09 for Group B) and the mean stone density with a mean of 979.95 ± 353.35 Hounsfield units (940.27 ± 362.97 for Group A and 1028.53 ± 337.96 for Group B) (Table 1). Most stones were single pelvic stones (Fig. 1). There was no significant difference between both groups regarding the parenchymal thickness and skin to stone distance which were measured by the CT scan (Table 1).

**Table 1:** Table comparing different demographic and stones parameters between both groups and their significance value

Preoperative parameters	Group A (*n* = 71)	Group A %	Group B (*n* = 58)	Group B %	*p* value
Gender (number)					0.83
Male	49	69.0%	39	67.2%	
Female	22	31.0%	19	32.8%	
Age (mean ± SD)	43.24 ± 13.03		44.60 ± 11.14		0.529
BMI (mean ± SD)	32.46 ± 6.27		31.36 ± 5.98		0.312
Previous ipsilateral renal surgery (number)	16	22.5%	15	25.9%	0.66
Previous ipsilateral ESWL (number)	14	19.7%	9	15.5%	0.535
Stone burden in cm (mean ± SD)	2.64 ± 1.15		2.78 ± 1.09		0.469
Stone density in hounsfield units (mean ± SD)	940.27 ± 362.97		1028.53 ± 337.96		0.159
Parenchymal thickness in mm (mean ± SD)	16.10 ± 6.70		16.22 ± 6.11		0.912
Skin to stone distance in cm (mean ± SD)	15.35 ± 8.40		14.45 ± 7.74		0.533

### Intraoperative parameters

Patients who underwent OSD PCNL had statistically significant shorter total fluoroscopy time, shorter fluoroscopy time during dilatation and shorter renal access time during dilatation than Group B (Table 2).

**Table 2:** Table comparing intra-operative and post-operative parameters across both groups and their significance value

Intra and post-operative parameters	Group A (*n* = 71)	Group A %	Group B (*n* = 58)	Group B %	*p* value
Dilatation time in seconds (mean ± SD)	34.41 ± 17.53		166.21 ± 82.12		< 0.001
Fluoroscopy time during dilatation in seconds (mean ± SD)	15.87 ± 6.87		98.45 ± 39.06		< 0.001
Total operative time in minutes (mean ± SD)	73.24 ± 31.84		97.93 ± 35.42		< 0.001
Total fluoroscopy time in minutes (mean ± SD)	3.69 ± 2.02		7.16 ± 3.29		< 0.001
Ureteral stents fixed [DJ stent] (number)	15	21.10%	16	27.60%	0.393
Stone-free patients (number)	55	77.50%	48	82.80%	0.216
blood transfusion (number)	3	4.20%	10	17.20%	0.015
Haemoglobin drop in g/dl (mean ± SD)	0.90 ± 0.99		1.34 ± 1.21		0.025
Creatinine elevation (mean ± SD)	0.00 ± 0.11		0.03 ± 0.17		0.293
Need for analgesia in number of doses (median [IQR])	3 (3)		4 (3)		0.011
Pain scale 0–10 (median [IQR])	4 (2)		4 (2)		0.043
Complications (number)	8	11.30%	22	37.90%	< 0.001
Pelvicalyceal perforation (number)	4	5.60%	1	1.70%	0.252
Post-operative stenosis (number)	0	0	1	1.70%	0.267
Post-operative fever	1	1.40%	6	10.30%	0.026
Urinary leakage (number)	1	1.40%	9	15.50%	0.003
Urinary leakage time in hours (median [IQR])	48.00 (48)		16 (36)		0.923
Need for delayed stenting (number)	1	1.40%	0	0	0.364
Hospitalization time in hours (mean ± SD)	36.61 ± 18.55		54.24 ± 33.60		0.001

### Post-operative parameters

Thirty patients encountered post-operative complications, 8 of them were from Group A while 22 patients were from Group B indicating a statistically significant difference in the rate of complications between both groups (*p* < 0.001). Most of these patients were complicated by fever (> 38 °C) which was managed conservatively with antibiotics, urinary leakage that was treated conservatively by observation and fluid restriction, and bleeding that required intravenous colloids or blood transfusion, and lastly pain that required opioid analgesics. All of those patients fell under the Clavien-Dindo classification grades I-II except for a single patient that required delayed stenting, who fell under grade IIIa. Another patient who underwent serial dilatation was complicated by infundibular stenosis that was diagnosed 4 months later by CT scan and required delayed stenting. There was a statistical difference in the number of patients who experienced post-operative urinary leakage between both groups with only a single patient in Group A, and nine patients in Group B (*p* = 0.003). Group A had a significant decrease in pain scores and fewer analgesic doses were needed than Group B (*p* = 0.006). Patients who underwent OSD have experienced less post-operative fever than patients who underwent serial dilatation. Group A showed shorter average post-operative hospitalization time. There was no statistically significant difference between both groups regarding stone-free rate for group A was 55/71 (77.5%), group B was 48/58 (82.8%) (Table 2).

## Discussion

Moufid et al. was the only literature found, comparing both types of dilatations that included tubeless PCNL which was conducted in 2012 (Table 3). Their study included a hundred consecutive patients randomly divided into two equal groups. The study found a significant difference in the dilatation time being 14 s shorter, total fluoroscopy time, total operative time being 20 min shorter, hospitalization time and procedural cost in favour of the OSD. There was no statistical difference in the haemoglobin drop, complications and success rate, hence it was concluded that tubeless OSD was the safer and more feasible [5]. The main limitations of that study were: All patients were in lateral decubitus position, which was not implemented, neither in our study, nor in the rest of the studies. Also, this study had deficient data regarding methodology of the study and selection criteria. In 2019, Sahin et al. compared between three types of dilatations; OSD, serial Alken and balloon dilatation in a retrospective study for all patients who had underwent PCNL. There was a remarkable statistical difference in the fluoroscopy time during dilatation being more than 5 s, shorter in OSD than the other two types of dilatations, also there was a significant difference in the haemoglobin drop of 0.52 mg/dl less in OSD compared to the other two types of dilatations. Operative time was 18 min shorter in OSD compared to serial dilatation [6]. Besides being a retrospective study the main drawback, also there was no data on whether patients with history of ipsilateral renal surgery or ESWL were included in this study or not [6]. Suelozgen et al. was the study that involved most patients in a retrospective non-controlled study involving only patients who had undergone OSD PCNL, who were 932 patients [7]. Despite having a large number of patients included in the study, this study’s limitations were mainly being a retrospective non-comparative study [7]. The rest of the studies mentioned [4, 8–13] were analysed in a systematic review and meta-analysis by Peng et al. in 2019, involving a total of 697 patients undergoing PCNL. The study reviewed seven prospective randomised controlled trials, comparing OSD with Serial dilatation in PCNL [3]. Aminsharifi et al. concentrated more on the safety regarding the renal scarring post-operative in both groups using Tc-99 m DMSA scan pre-operative and four weeks post-operative, in which a more significant renal scarring was present in the OSD group rendering it for possibly being less safe than the serial dilatation, despite having less fluoroscopy and operative time [17]. One-Shot dilatation is more susceptible to renal parenchymal scarring than serial dilatation as concluded by Aminsharifi et al. [10]. Frattini et al. and Srivastava et al. compared between more than two types of dilatations, yet still OSD was found to be superior than others, being safer and more feasible [4, 13, 15]. Amajad et al. and Falahatkar et al. compared OSD and serial dilation but the main limitation was small sample size [14, 16].

**Table 3:** Comparison between the methodologies of similar studies

Author	Year	Location	Number of cases		Gender (M/F)		Age		Stone size		Study type
			OSD	Serial	OSD M/F	Serial M/F	OSD	Serial	OSD	Serial	
Sahin et al. [6]	2019	Turkey	61	66	40/21	48/18	46.5	48.9	2.07	2.32	Retrospective
Moufid et al. [5]	2012	Morocco	50	50	NR	NR	NR	NR	NR	NR	Prospective
Frattini et al. [15]	2001	Italy	26	27	15/12	17/9	59	54	2.3 ± 0.7	2.9 ± 0.9	Prospective
Amjad et al. [14]	2008	Iran	17	14	10/7	12/2	42	44	3.7 ± 1.0	3.2 ± 1.1	Prospective
Falahatkar et al. [16]	2009	Iran	102	112	56/46	62/50	57	51	3.9 ± 1.6	3.4 ± 1.2	Prospective
Aminsharifi et al. [17]	2011	Iran	29	19	19/10	9/10	44.1	42.5	2.7 ± 1.0	3.7 ± 1.3	Prospective
Nour et al. [11]	2014	Egypt	24	25	17/7	16/9	43.8	38.2	3.0 ± 0.7	3.0 ± 0.7	Prospective
Hosseini et al. [12]	2014	Iran	31	31	22/9	18/13	3.7	3.7	2.0 ± 0.4	1.7 ± 0.4	Prospective
Srivastava et al. [13]	2017	India	120	120	59/61	62/58	38.9	40.1	NR	NR	Prospective
Suelozgen et al. [7]	2017	Turkey	932	0	558/374	0	48.9	N/A	NR	N/A	Retrospective
This study	2020	Egypt	71	58	49/22	39/19	43.2	44.6	2.6 ± 1.1	2.8 ± 1.1	Prospective

*NR* not reported, *N/A* not available

Although most of the results discussed previously in all of the studies including our study concluded that OSD is safer and more feasible, none of the studies except our study compared the dilatation techniques in Tubeless PCNL, and only a few studies have included patients with history of previous ipsilateral renal surgery or ESWL. Only a single study has ever studied the risk of renal scarring by renogram in both types of dilatations. None of the mentioned studies have specifically included Tubeless PCNL in their studies. More studies are needed to compare between other types of dilatations such as balloon dilatation. Therefore, more randomised controlled trials with high-volume single centre, preferably same surgeon, or surgeons at the same level of expertise to avoid bias, is recommended to draw a final conclusion to the urological practice.

## Conclusion

In our study, one-shot dilatation showed shorter dilatation time, fluoroscopy time, operative time, fluoroscopy during dilatation time, and hospitalization time. Also, it had less risk of haemorrhage, less analgesic requirements, low complications rate, and post-operative fever than serial dilatation, so it is deemed to be safer and more feasible than serial dilatation in tubeless PCNL.

## Data Availability

All data and materials as well as software application are available and the transparency on data statements from all authors are in the mauscript as appropriate.
